# Reduction of plasma glutathione in psychosis associated with schizophrenia and bipolar disorder in translational psychiatry

**DOI:** 10.1038/tp.2017.178

**Published:** 2017-08-22

**Authors:** L G Nucifora, T Tanaka, L N Hayes, M Kim, B J Lee, T Matsuda, F C Nucifora Jr, T Sedlak, R Mojtabai, W Eaton, A Sawa

**Affiliations:** 1 Department of Psychiatry and Behavioral Sciences, Johns Hopkins University School of Medicine, Baltimore, MD, USA; 2 Department of Mental Health, Johns Hopkins University Bloomberg School of Public Health, Baltimore, MD, USA

## Abstract

The establishment of mechanism-driven peripheral markers is important for translational psychiatry. Many groups, including ours, have addressed molecular alterations in peripheral tissues in association with symptomatic changes in major illnesses. Oxidative stress is implicated in the pathophysiology of schizophrenia (SZ) and bipolar disorder (BP) through studies of patient peripheral tissues and animal models. Although the relationship between peripheral changes and brain pathology remain elusive, oxidative stress may bridge such translational efforts. Nonetheless, the molecular substrates of oxidative stress are not well defined in mental conditions. Glutathione (GSH) is a non-enzymatic antioxidant that eliminates free radicals, and has been suggested to have a role in SZ. We performed a cross-sectional study of 48 healthy controls (CON), 52 SZ patients and 62 BP patients to compare the levels of peripheral GSH by a biochemical enzyme assay. We show a significant reduction of plasma GSH in both SZ and BP patients compared with CON. We evaluated possible influences of clinical characteristics on the level of GSH in SZ and BP. A decrease in GSH level correlated with Positive and Negative Syndrome Scale (PANSS) total and positive scores for SZ and correlated with the PANSS general for BP. Taken together, we provide evidence that SZ and BP display a common molecular signature in the reduction of peripheral GSH in the psychosis dimension.

## Introduction

One of the challenges that face psychiatry today is the absence of mechanistic-driven diagnostic biomarkers.^1^ Psychiatric diagnosis is still made based on symptomatology and lacks any molecular foundation. The inability to directly access the brain limits the development of diagnostic measures in psychiatry. As a result, peripheral tissues such as blood and cerebrospinal fluid (CSF) from patients are now being used to study the underlying molecular mechanisms of psychiatric disease. The molecular mediators identified from these studies could serve as potential biomarkers.

Another current challenge in psychiatry is the validity of categorizing mental conditions into separate diseases.^2^ Modern psychiatry, influenced by the views of Emil Kraepelin, distinguishes schizophrenia (SZ) from bipolar disorder (BP). However, in the past decade, multiple lines of scientific evidence challenged this traditional dichotomy. Psychiatric genetics provides compelling data that SZ and BP share common genetic etiologies.^3, 4^ Neuropsychology suggests that cognitive deficits in SZ and BP are similar, although the severity is worse in SZ compared with BP.^5, 6^ Brain imaging studies demonstrated an overlap of neuroanatomical changes, such as gray matter reductions in both SZ and BP.^7^ It remains elusive whether common key molecular mediators exist in the pathophysiology of both SZ and BP, and if they connect shared genetic risks with gross phenotypic manifestations at the anatomical and clinical levels.

Oxidative stress has been studied in the context of severe mental disorders.^8, 9, 10, 11^ Oxidative stress occurs as the result of an accumulation of free radicals generated by normal metabolism and various environmental exposures to elicit cellular dysfunction.^12, 13^ Enzymatic and non-enzymatic antioxidants eliminate these free radicals to achieve a balance between free-radical generation and extinction.^12, 13^ These antioxidants include superoxide dismutases (SODs), catalase and glutathione (GSH).^14, 15, 16, 17, 18, 19, 20^ GSH is a representative non-enzymatic antioxidant that exists at low intracellular levels and functions as an important regulator of redox balance and oxidative stress.^21, 22^

It still remains elusive whether and how changes in peripheral oxidative stress markers reflect an alteration of these molecules in the brain. Some reports suggest the existence of oxidative stress in patients’ brains in parallel with peripheral tissues.^14, 23, 24, 25, 26^ In preclinical studies, excess oxidative stress is consistently observed in the brains of animal models with etiological triggers relevant to mental illness, in particular SZ. These models include those of neonatal hippocampal lesion, expression of mutant DISC1 protein and genetic deletion of the key GSH-synthesizing enzyme.^27, 28, 29, 30, 31^ Most importantly, prophylactic administration of antioxidants can ameliorate behavioral deficits in adulthood in animal models.^28, 31^ Thus, a possible mechanism of how excess oxidative stress and possibly GSH cascades may underlie behavioral changes has been explored. Parvalbumin-positive interneurons in the cortex and hippocampus are believed to have a key role in cognition and other higher brain functions,^32, 33, 34^ and these neurons are particularly sensitive to oxidative stress.^27, 30, 35^ Thus, these specific interneurons may be a candidate substrate of oxidative stress in the pathophysiology of mental illness.

Guided by many promising preclinical studies that potentially link GSH and oxidative stress with the pathophysiology of SZ, multiple groups have also conducted clinical studies and measured GSH levels in peripheral blood of patients with SZ. Although many studies report the reduction of GSH in tissues of SZ patients, there are still some inconsistencies among these reports.^36, 37, 38, 39, 40, 41, 42, 43, 44, 45^ This is partly because some studies were performed in a small sample size, and further confirmation is needed.^36, 37, 38, 39, 40, 41, 42, 43, 44, 45^ An alternative approach is to explore correlations between symptoms and GSH.^46^ Contrary to the abundance of studies in SZ, only three studies measured GSH in BP, which had conflicting results.^47, 48, 49^ Given that SZ and BP likely share common pathophysiological mechanisms, it is an important question whether or not GSH is altered in both SZ and BP. Such commonality and specificity of molecular changes among mental illness are to be investigated from the viewpoints in translational psychiatry.

In the present study, we address the significance of peripheral GSH in SZ and BP. We recruited BP patients that exhibited at least one psychotic episode because they share some of the neurocognitive or neuroanatomical deficits observed in SZ.^50, 51^ First, we examined the levels of plasma GSH in patients with SZ and BP compared to healthy controls (CON). Second, we correlated peripheral GSH levels with clinical symptoms and neurocognitive functions. Finally, we assessed the effect of types of medication, drug adherence, duration of illness and previous psychiatric hospitalization on the level of GSH in SZ and BP.

## Materials and methods

### Participants

All patients (*n*=114) were recruited as part of the Monitoring Recovery from an Episode of Severe Mental Illness (RECOVERY) study in the greater Baltimore–Washington, D.C. area from two cohorts admitted between August 2008 and December 2012 from the Johns Hopkins Hospital Community Psychiatry Program and the Johns Hopkins Bayview Medical Center Community Psychiatry Program. Participants had DSM-IV clinical diagnoses of SZ, schizoaffective disorder, schizophreniform disorder, BP (type I or II), delusional disorder or psychotic disorder not otherwise specified. All participants experienced at least one psychotic episode and were able to speak and understand English. The participants enrolled were screened for heart disease, diabetes and kidney disease and were not taking supplements. The study was approved by the Institutional Review Board of the Johns Hopkins Bloomberg School of Public Health. We obtained written informed consent from all participants after a complete description of the study. The CON (*n*=48) were obtained from the Prevention Research Center (PRC) trial and The Johns Hopkins Schizophrenia Center (JHSZC). A second control cohort was collected from the Baltimore Epidemiologic Catchment Area (ECA) follow-up study but was not used in the final study. None of the CON met the DSM defined criteria for psychiatric disorders.

Psychiatric symptoms were assessed in the patients using the Positive and Negative Syndrome Scale (PANSS).^52^ The PANSS is a 30 item rating scale designed to measure symptomatology and is completed by a trained interviewer after a semi-structured interview. The PANSS has a total and three subscale scores: positive, negative and general psychopathology. Cognitive function was assessed using the Brief Assessment of Cognition in Schizophrenia (BACS).^53^ The BACS composite score is calculated by averaging six subdomains: working memory, motor speed, verbal fluency, attention and speed of information processing, and executive function. We used the BACS composite score as an overall indicator for neurocognitive function. We also collected information on smoking status (smoker/non-smoker), types of medications (antipsychotics/mood stabilizers/antidepressants/minor tranquilizers), adherence to mediations (everyday/almost everyday/most days/hardly ever/never), duration of illness, years of education and previous psychiatric hospitalization. All patients completed clinical assessments except one SZ patient who refused the PANSS and BACS assessments, and one BP patient who refused the PANSS assessment. The CON did not receive any BACS assessment.

### Measurement of GSH in plasma

Peripheral whole-blood samples were collected from each participant by venous puncture (Supplementary Table 1). Blood collection was not taken at the time of diagnosis. The mean delay between blood collection and diagnosis is not available. The Johns Hopkins Genetic Resources Core Facility processed samples from all cohorts; samples that arrive before 1500 hours are processed within 2 h and samples after are processed immediately the next morning (~16–18 h). Plasma was isolated by centrifugation at room temperature at 1960 × *g* for 15 min, aliquoted and stored at−80 °C. Total GSH (the sum of GSH and glutathione disulfide (GSSG)) was measured in plasma using modifications of the Tietze method.^54, 55^ Specifically, plasma samples were deproteinated by adding 50% v/v of freshly prepared 10% metaphosphoric acid (MPA), centrifuging at 2000 × *g* for 2 min and then the supernatant stored at −20 °C until assaying. For the assay, 5 μl of freshly prepared 4 m triethanolamine was added to 100 μl of MPA-treated plasma. GSH was then measured according to kit specifications (Cayman Chemical, Ann Arbor, MI, USA, 703002). Each plasma sample was measured in duplicate and reported as the average of the two values. We ran a standard curve from 1  to 0.016 μm that maintained linearity with a slope of 26±5 and an *R*^2^ of 0.998±0.001 (Supplementary Figure 1). The rate of increase in absorbance at 415 nm, which measures the reduction of 5–5′-dithiobis (2-nitrobenzoic acid) by GSH, reflects the total GSH content. The concentration of total GSH in plasma was reported as μm. We describe total GSH simply as GSH.

### Statistical analysis

Statistical analyses were performed using R version 3.3.0 for Windows (The R Foundation for Statistical Computing, Vienna, Austria). Group comparison of demographic and clinical data were conducted using Welch’s two sample *t*-test for continuous variables, except for age in which one-way ANOVA was used. Comparisons of categorical data were performed using Fisher’s exact test with Monte Carlo simulation *N*=1 000 000. Direct comparisons of demographic and clinical data between healthy CON vs SZ/BP were accomplished using Welch’s two sample *t*-test.

The Shapiro–Wilk normality test was performed on GSH and log GSH levels, and showed non-normal distributions for both SZ and BP patients, therefore subsequent correlation analysis was performed on total GSH levels. To compare GSH levels in plasma of patient and CON groups, we used ANCOVA analysis with adjustment for age, sex, ethnicity and smoking status to determine differences among the three groups. To determine differences between groups, we performed a *post hoc* pair-wise (Tukey–Kramer) comparison.

Correlation between GSH levels and PANSS (total, positive, negative and general) or BACS composite score was performed using Spearman’s rank correlation coefficient. Partial correlations were further tested to control for age, smoking status, gender and ethnicity. We also performed multiple regression analyses to evaluate the clinical characteristics (severity of symptoms, neurocognitive functions, previous hospitalization and comorbid health conditions) on total GSH level in SZ and BP. We performed the multiple regression analyses with and without the interaction term (clinical characteristics × diagnosis). All data were expressed as mean±standard deviation (s.d.). Statistical significance was defined as *P*<0.05.

## Results

### Study population and neuropsychiatric assessment

In the present report, we studied patients with SZ, those with BP, and CON. These three groups were not fully matched with regard to age, gender, ethnicity and smoking status (Table 1). The age of patients was significantly higher than CON. The distributions of sex, ethnicity and smoking status were significantly different between SZ and BP (Table 1). Therefore, we incorporated age, sex, ethnicity and smoking status in our regression model to adjust for these variables. We did not see a significant difference between SZ and BP in the duration of illness calculated at the age of initial psychiatric contact. The rate of past hospitalization was similar in SZ and BP. As expected, SZ patients received more antipsychotic medication compared to BP (*P*<0.001), and BP patients received more mood stabilizers than SZ (*P*<0.001). There was also increased use of benzodiazepines in BP compared with the SZ patients (*P*<0.016). There was no significant difference in use of antidepressant or in adherence to the medications between SZ and BP. PANSS total scores were significantly lower in BP than SZ, and BACS composite scores were significantly higher in BP than SZ (*P*<0.001).

### Measurement of GSH: influences of tissue storage

Storage duration may affect the levels of metabolites in biospecimens. To address this question, we examined the influence of total GSH level on storage time (in months) in plasma samples from the RECOVERY, PRC and JHSZC cohorts as well as the ECA cohort. The RECOVERY, PRC and JHSZC plasma samples had a storage time of <100 months at the time of the biochemical experiment, whereas the ECA cohort had a storage time >100 months. When we included all cohorts (RECOVERY, PRC, JHSZC and ECA) independent of storage time, we obtained similarly trended results in comparison to the analysis performed using plasma samples with a storage time of <100 months (RECOVERY, PRC and JHSZC). The data showed that storage time did not have a significant influence on total GSH level. This result was not affected by the inclusion of the ECA cohort.

Although storage time did not significantly influence total GSH level, we did observe a slight decrease in GSH level with increased storage time when the ECA cohort was included. To take a conservative approach, we chose to only include plasma samples with a storage time of <100 months. Therefore, the final study included plasma samples from the RECOVERY, PRC and JHSZC cohorts.

**Figure 1:**
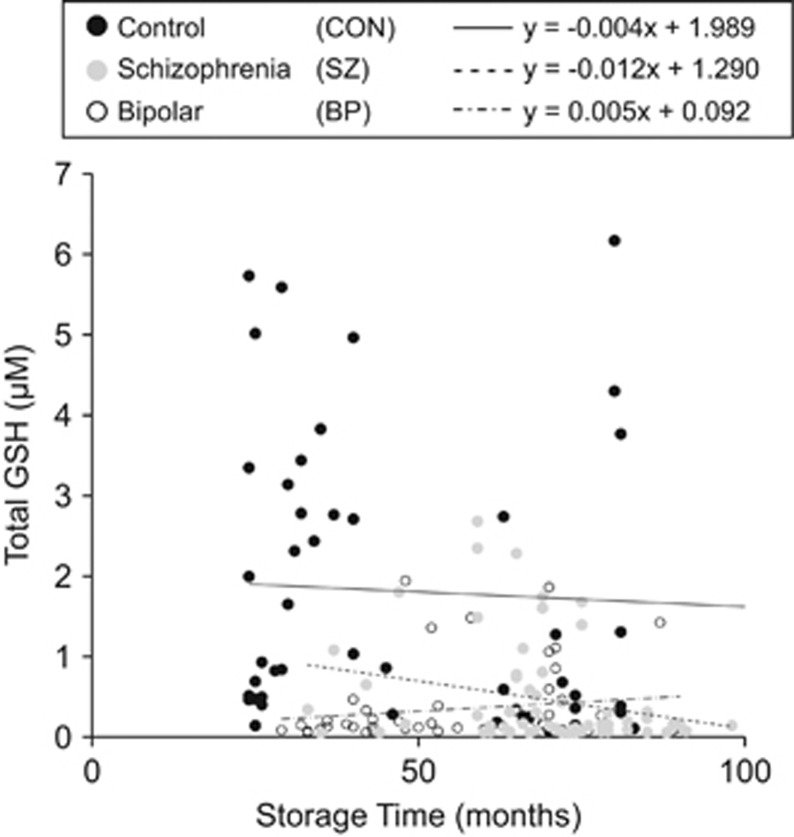
Effect of total glutathione (GSH) levels upon storage duration of control and patient plasma samples. The levels of GSH are plotted against storage time for healthy control (CON) (●), bipolar disorder (BP) (○) and schizophrenia (SZ) (
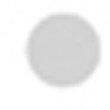
) plasma samples. All CON and patient plasma samples had a storage time of <100 months. A trend line of the data was obtained for each sample (CON (–), *y*=−0.004x+1.989; BP (–·–·–) *y*=0.005*x*+0.092; SZ (– – –) *y*=−0.012*x*+1.290). The variability of slopes across the three sample populations indicates storage time is not a significant influencing factor on total GSH levels and is more an effect of the random overall distribution of GSH levels within each group.

The total GSH level vs storage time in months from those cohorts are shown in Figure 1, a best fit line and slope were reported for each CON, BP and SZ group. The slopes for CON and SZ plasma samples were slightly negative showing an overall decrease in GSH level with increase in storage time (Figure 1). Conversely, the slope for BP samples was positive indicating an increase in GSH levels with increased storage time (Figure 1). A lack of a consistent trend in effect of GSH level over storage time across the three groups indicates that storage time is not an influencing factor in this study and reflects the overall distribution of GSH levels. This is also confirmed by the fact that our results were not affected by including the older control ECA cohort samples.

### Comparison of GSH levels in plasma in patient and control groups

**Figure 2:**
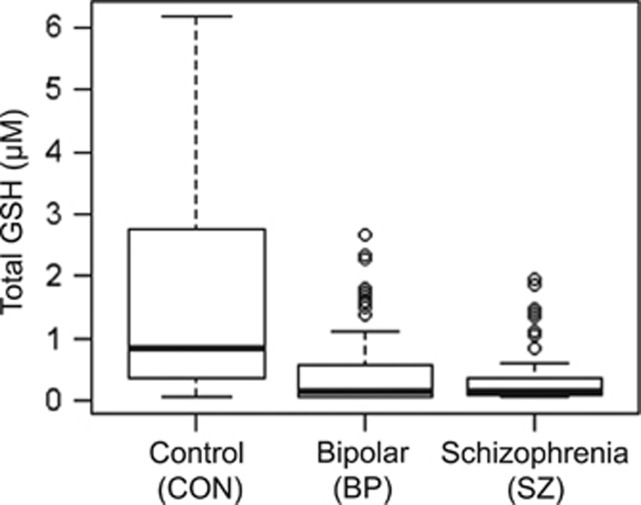
Reduction of plasma glutathione (GSH) levels in schizophrenia (SZ) and bipolar disorder (BP) compared with healthy controls (CON). The levels of plasma GSH were measured in CON, SZ and BP. The GSH levels in CON, SZ and BP are depicted as box-plots. The GSH level was significantly lower in both patient groups compared with CON (CON, 1.737±1.776; SZ, 0.365±0.478; BP, 0.476±0.655; CON vs SZ, *P*<0.001; CON vs BP, *P*<0.001).

We examined the levels of GSH in plasma from the three groups (CON, SZ and BP). We observed decreased GSH in SZ compared to CON (CON, 1.736±1.766; SZ, 0.365±0.478) (Figure 2). We also observed decreased GSH in BP compared to CON (CON, 1.736±1.766; BP, 0.476±0.655) (Figure 2). Levels of GSH in SZ and BP patients (SZ, 0.365±0.478; BP, 0.476±0.655) were comparable.

We used ANCOVA analysis with adjustment for age, sex, ethnicity and smoking status to observe differences among the three groups. We observed a significant difference among the groups (*P*<0.001). We performed a *post hoc* pair-wise (Tukey–Kramer) comparison adjusting for age, sex, ethnicity and smoking to determine differences between groups. We obtained a significant decrease between SZ and CON groups (*P*<0.001), and BP and CON groups (*P*<0.001). There was not a significant difference between the SZ and BP groups (*P*=0.802).

Although we adjusted for age in our ANCOVA analysis, we were interested in observing the effect of total GSH levels upon age. We plotted total GSH level vs age for CON, BP and SZ (Supplementary Figure 2). As observed, the trend line for the CON was consistently approximately twofolds higher than the corresponding SZ and BP trend line when considering total GSH level over the entire age distribution.

### GSH levels and clinical characteristics

Next, we determined whether the levels of peripheral GSH were correlated with PANSS scores or BACS scores in SZ and BP, respectively, correcting for age, sex, ethnicity and smoking (Table 2). We observed significant correlation of the PANSS total (*P*=0.033) and PANSS positive (*P*=0.013) scores with total GSH level within the SZ patient population. We also observed significant correlation between the PANSS general score (*P*=0.035) and total GSH level within the BP patient population. We did not observe significant correlation between total GSH level and BACS scores in SZ and BP.

Finally, we performed multiple linear regression analyses to assess the effect of each clinical characteristic of severity of disease, previous hospitalization, illness duration and medication adherence on the level of total GSH. We found no significant effect on total GSH level from any of these clinical characteristics.

## Discussion

The present study demonstrates a significant reduction in the level of plasma GSH in both SZ and BP patients compared to CON. Our data, from a reasonably large sample size, validates the significance of GSH deficits in SZ pathology, which has been suggested previously.^36, 37, 38, 39, 40, 41, 42^ In contrast, it has been unclear whether GSH had a role in BP, because only three previous studies showed conflicting results.^47, 48, 49^ This study provides evidence that GSH deficits are also present in patients with BP, indicating that the reduction of peripheral GSH is a common molecular signature of SZ and BP. Given that the BP patients in the present study experienced psychosis, GSH may be involved in psychosis-associated pathophysiology that is shared by both SZ and BP.

As we propose that this reduction is related to psychosis, we do not exclude that this reduction may be associated with other conditions such as neurodegenerative disorders. This study did not include the quantification of GSH levels in BP without psychosis, but we view it is an important topic to investigate in a future study. Although blood collection was taken after diagnosis and not during a period of psychosis, we do not believe the reduction of total GSH level is a direct indicator of a patient’s psychotic state, but is associated with a predisposition to psychosis. Finally, we report total GSH values in the present study, and acknowledge the significance in understanding the contribution of the reduced (GSH) and oxidized (GSSG) forms of GSH to the overall blood level observed and will address this in the future.

We further addressed how the GSH changes are related to clinical features. The levels of plasma GSH were significantly correlated with PANSS scores in both SZ and BP, which may support the idea that the changes in the GSH levels are associated with the psychosis dimension commonly underlying these two diseases. The type of medication administered to each patient group was significantly different. Thus, the reduction of GSH may be an intrinsic pathophysiological change common to SZ and BP rather than an effect of medication. By studying SZ, BP and CON in the same study, we are now able to propose an important role for GSH in both SZ and BP.

Establishing high-throughput, objective biomarkers is important to the field of translational psychiatry. Towards this goal, molecules that are associated with pathophysiological mechanisms (for example, pathophysiological mediators altered in the disease conditions) are promising candidates for such biomarkers. More practically, such candidates are scalable in measurement, with reasonable sensitivity and specificity. Although it is too early to state that GSH is a promising biomarker under the criteria, the data in the present study provide an optimistic perspective that molecules in GSH signaling (even it is not GSH itself) may be candidates for biomarkers in the future.

Although GSH is scalable in measurement by using peripheral blood, it remains elusive how the changes in peripheral GSH reflect brain pathology. The variability in the levels of peripheral GSH, although the absolute differences in GSH levels between patient and control groups are significant, is high. Measurement of blood GSH may be a useful indicator to estimate psychosis but this cannot stand as a biomarker by itself. Instead, similar to the C-reactive protein, which is still regarded as useful in monitoring the pathological status of immune-related conditions (for example, rheumatoid arthritis), we propose that GSH can be used as a candidate objective marker for mental disorders, in combination with other markers. These markers have smaller variability with their sensitivity and specific complementary to those of GSH that are associated with psychotic status in general.

We acknowledge potential limitations in the present study design. First, the present study was designed in a cross-sectional manner. Thus, it is difficult to determine a causal relationship between the levels of GSH and clinical outcomes. A prospective study that measures GSH and its relationship with clinical features is crucial to assess the potential clinical utility of GSH in these pathological conditions. Second, the participants in this study were recruited in the context of public health and as a result medical histories, including information on liver disease, were limited. All patient participants were screened for heart conditions, diabetes and kidney disease. Third, fasting state and age are not perfectly controlled, and there is variability in blood processing time and time of blood collection following diagnosis; nevertheless, as these confounding factors underlie all groups, it is unlikely that these factors affect the conclusion. Fourth, we focused on patients with severe mental disorders who experienced at least one psychotic episode and the measures were limited to common assessment tools that can be applied to both SZ and BP. Therefore, we did not rate BP patients with the Young Mania Rating Scale or the Montgomery Asberg Depression Rating Scale.^56, 57^ BACS does not include the assessment for some cognitive constructs, such as ideational fluency and visual memory, which are known to be impaired in patients with SZ and BP.^5^ Thus, the negative results in the correlation of the GSH levels and the BACS composite scores do not necessarily mean that GSH and cognitive deficits are unrelated in the diseases. For example, neurocognitive batteries such as the calibrated ideational fluency assessment word/design fluency or the brief visuospatial memory test will complement this limitation in future studies.

In previous reports, we observed molecular changes in SOD1 and inflammatory-related molecules in CSF only in the early phase of disease but not in the chronic phase.^19, 20^ Changes in many of these molecules were consistently observed in another cohort for early psychosis.^58^ In contrast, the present study highlights the change of an oxidative stress-associated molecule in the chronic stage of SZ and BP. These results imply two different but potentially overlapping interpretations. First, a reduction in plasma GSH may be an enduring trait after onset of disease, which contrasts with the reduction of SOD1 in CSF that preferentially manifested in the early stages of psychosis.^19^ Second, reduction in plasma GSH may represent a general risk of mental illness, such as SZ and BP, which may exist even before the onset of disease. Future studies in a longitudinal design may provide an answer to the first question. To address the second question, a systematic study of plasma GSH that includes high-risk cohorts and first-degree relatives will be useful. In addition, we may need to compare the potential differences between data from different tissues (for example, plasma and CSF). Although peripheral biomarkers are important in translational and clinical psychiatry, it remains mechanistically unclear how molecular changes in the blood and brain are related. Therefore, preclinical animal models may aid in further understanding this link and the underlying mechanisms of oxidative stress in disease.

## Figures and Tables

**Table 1:** Clinical and demographic characteristics (plasma storage time<100 months)

*Characteristics*	*CON (*N*=48)*	*SZ (*N*=52)*	*BP (*N*=62)*	P*-value*^a^	*CON* *vs* *SZ* P*-value*^b^	*CON vs BP* P*-value*^b^
Age	29.02±7.10	42.46±10.52	41.13±10.75	<0.001*	<0.001*	<0.001*
Gender (male/female)	29/19	28/24	18/44	0.002*	0.549	0.002*
Ethnicity^c^ (AA/C/H/A/other)	31/16/0/0/1	35/12/1/0/4	22/30/1/1/8	0.004*	0.332	0.008*
Smoking^d^ (smoker/non-smoker/unknown)	13/35/0	31/5/16	42/4/16	<0.001*	<0.001*	<0.001*
Duration of Illness (years)^e^	—	14.10±12.71	11.07±11.69	0.195	—	—
Previous hospitalization (yes/no)	—	41/11	46/16	0.660	—	—
Antipsychotics (yes/no)	—	42/10	25/37	<0.001*	—	—
Mood stabilizers (yes/no)	—	7/45	32/30	<0.001*	—	—
Antidepressant (yes/no)	—	16/36	23/39	0.554	—	—
Benzodiazepines (yes/no)	—	3/49	14/48	0.016*	—	—
Adherence to medication^f^	—	34/9/4/0/1/4	36/13/7/1/3/2	0.703	—	—
PANSS total score^g^	—	65.29±21.44	52.77±15.15	<0.001*	—	—
BACS composite score^h^	—	30.96±7.51	36.70±7.90	<0.001*	—	—

Abbreviations: BACS, Brief Assessment of Cognition in Schizophrenia; BP, bipolar disorder; CON, healthy control; PANSS, Positive and Negative Syndrome Scale; SZ, schizophrenia.

a Welch’s two sample *t*-test is used for continuous variables, except for age in which one-way ANOVA is used. Fisher’s exact test is used for categorical variables with Monte Carlo simulation *N*=1 000 000.

b Welch’s two sample *t*-test was used to compare control vs SZ/BP.

c African American/Caucasian/Hispanic/Asian/other.

d Missing smoking status is considered unknown.

e Information for 1 SZ and 1BP patient is missing.

f Everyday/almost everyday/most days/hardly ever/never/not available.

g One patient with SZ and 1 patient with BP refused for PANSS assessment.

h One patient with SZ refused to take BACS assessment.

*P<0.05.

**Table 2:** Partial correlation of GSH levels with PANSS and BACS scores in SZ and BP, corrected for age, sex, ethnicity and smoking

	ρ^c^	P-*value*
*SZ*^a^*(*n*=52)*		
PANSS total	−0.311	0.033*
PANSS positive	−0.359	0.013*
PANSS negative	−0.203	0.172
PANSS general	−0.262	0.075
BACS average	0.245	0.097
		
*BP*^b^*(*n*=62)*		
PANSS total	−0.204	0.127
PANSS positive	−0.140	0.298
PANSS negative	−0.107	0.427
PANSS general	−0.279	0.035*
BACS average	−0.062	0.645

Abbreviations: BACS, Brief Assessment of Cognition in Schizophrenia; BP, bipolar disorder; GSH, glutathione; PANSS, Positive and Negative Syndrome Scale; SZ, schizophrenia.

a One SZ patient refused to take PANSS and BACS assessment.

b One BP patient refused to take PANSS.

c Shapiro–Wilk normality test was performed on GSH and log GSH levels, and showed non-normal distributions in both SZ and BP patients, so Spearman’s rank correlation coefficient was used for GSH levels, with partial correlation corrected for age, sex, ethnicity and smoking status. *P<0.05.
