# Supplementary figures and images for: Reduction of plasma glutathione in psychosis associated with schizophrenia and bipolar disorder in translational psychiatry
Source: Transl Psychiatry. 2017 Aug 22;7(8):e1215–. doi: 10.1038/tp.2017.178 (PMC5611744; doi:10.1038/tp.2017.178)

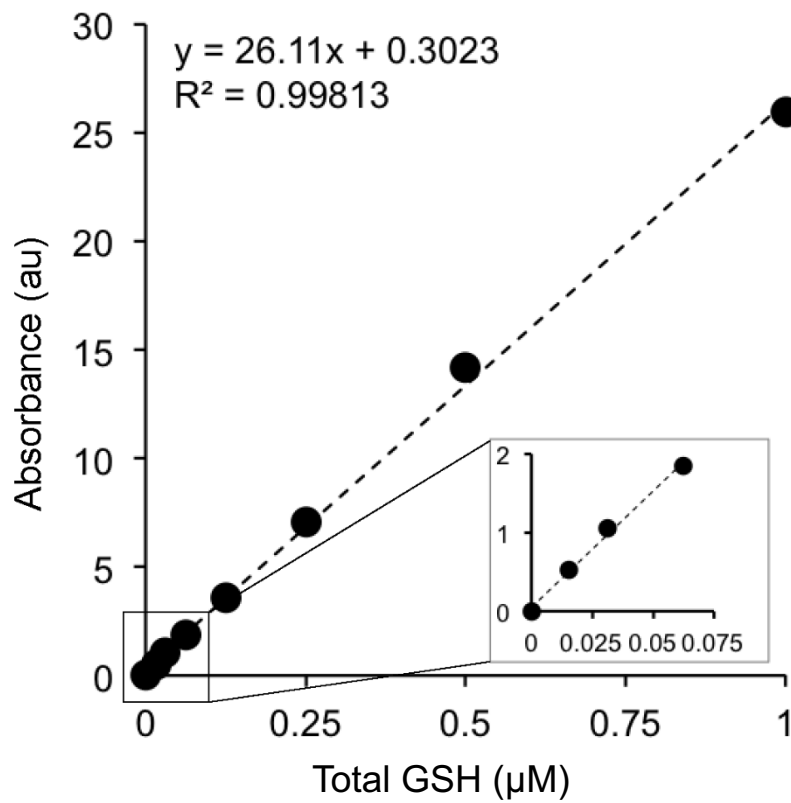

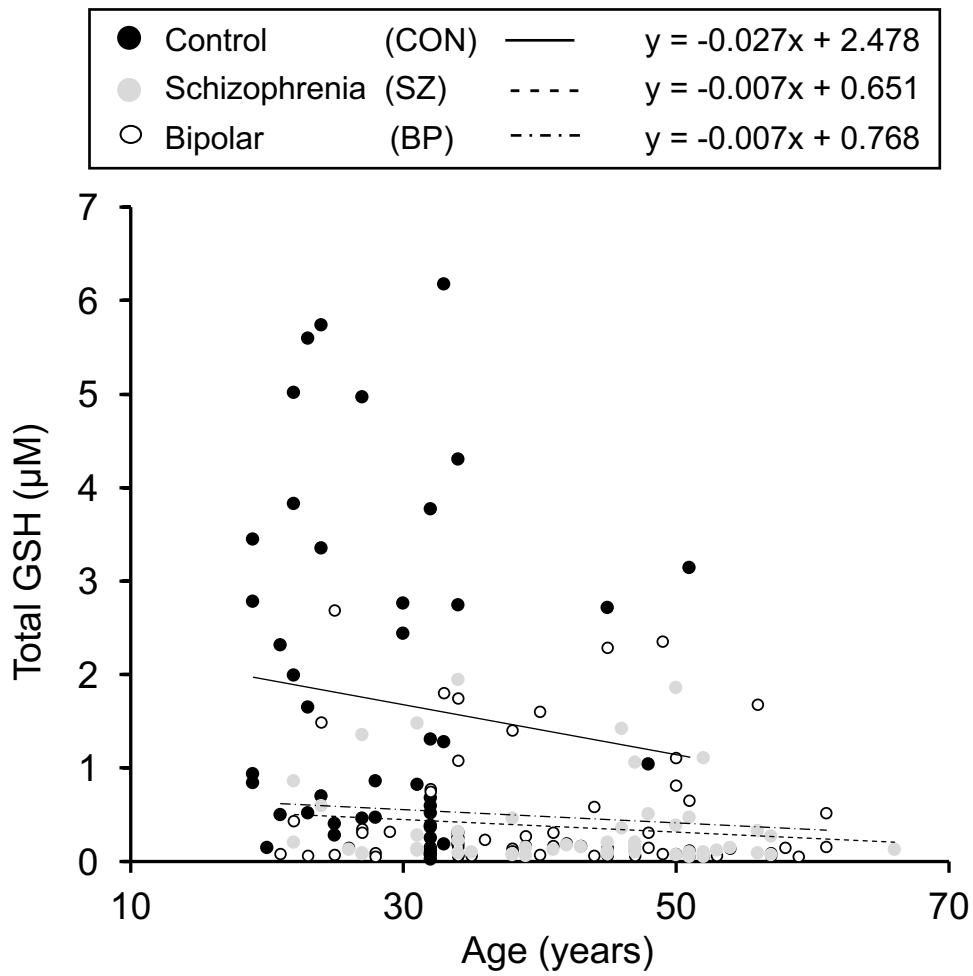

Supplement: Supplementary Figures [file tp2017178x1.pdf]
