# Supplementary material for: Reduction of plasma glutathione in psychosis associated with schizophrenia and bipolar disorder in translational psychiatry
Source: Transl Psychiatry. 2017 Aug 22;7(8):e1215–. doi: 10.1038/tp.2017.178 (PMC5611744; doi:10.1038/tp.2017.178)
Supplement: Supplementary Figure Legends [file tp2017178x2.docx]

**Supplemental Figure Legends**

**Supplemental Figure 1. Extended Standard Curve for the determination of GSH.** Each point on the plot is an average of two independent measurements. The standard curve included GSH concentrations extending from 0.016 µM to 1 µM and maintained linearity with a slope of 26 ± 5 and an R2 of 0.998 ± 0.001.

**Supplemental Figure 2. Effect of total GSH level upon age.** The total GSH levels were plotted against age for CON (●), BP (○) and SZ (●) plasma samples. A trend line of the data was obtained for each sample (CON ( ), y = -0.027x + 2.478; BP ( ) y = -0.007x + 0.651; SZ, (- - -) y = -0.007x + 0.768). An overall decrease of total GSH level was observed with age for both CON and BP/SZ participants. The trend line for the CON were approximately 2-fold higher than the corresponding trend line for SZ and BP across the entire age distribution.
