# Supplementary material for: Reduction of plasma glutathione in psychosis associated with schizophrenia and bipolar disorder in translational psychiatry
Source: Transl Psychiatry. 2017 Aug 22;7(8):e1215–. doi: 10.1038/tp.2017.178 (PMC5611744; doi:10.1038/tp.2017.178)
Supplement: Supplementary Table 1 [file tp2017178x3.docx]

**Supplemental Table 1** | Conditions of sample plasma collection across cohorts

| **Cohort** | **Anti-coagulant** | **Time Collected** | **Year Collected** | **Time to Processing** | **Fasting** | **Storage before processing** | **Storage after processing** |
| --- | --- | --- | --- | --- | --- | --- | --- |
| ECA^a^ | K_2_EDTA | n/a | 1/15/2004  –  4/18/2005 | Within 12 h | No | 4ºC | -80°C |
| PRC^b^ | K_2_EDTA | n/a | 4/3/2009  –  1/7/2011 | n/a | n/a | R.T. | -80°C |
| Recovery^c^ | K_2_EDTA | 7:30 am-12 pm | 1/29/2008 – 10/29/2013 | Within 12 h | Yes | R.T. | -80°C |
| JHSZC^d^ | K_2_EDTA | 9 am-4 pm | 2/26/2006 – 3/13/2014 | 2 h or 16-18 h | No | R.T. | -80°C |

a, 45 CON subjects (not used in this study).

b, 21 CON subjects.

c, 52 SZ subjects and 62 BP subjects.

d, 27 CON subjects.
